# The Relationship Between TRIPS, MINT, SNAPPE-II Scores, and Mortality in Newborns Transported Within the First 24 h of Birth

**DOI:** 10.3390/jcm15052062

**Published:** 2026-03-08

**Authors:** Mehtap Durukan Tosun, Nihan Ozel Ercel, Istemi Han Celik, Fatih Isleyen, Fatma Pinar Tabanlı, Ahmet Yagmur Bas, Nihal Demirel

**Affiliations:** 1 Department of Paediatrics, Neonatology, Mersin City and Education Hospital, Mersin 33800, Turkey; 2 Department of Biostatistics and Informatics in Medicine, Mersin University Faculty of Medicine, Mersin 33110, Turkey; nihanozel88@gmail.com; 3 Department of Paediatrics, Neonatology, University of Health Sciences, Etlik City Hospital, Ankara 06170, Turkey; istemihancelik@gmail.com; 4 Department of Paediatrics, Neonatology, Sanliurfa Education and Research Hospital, Sanliurfa 63250, Turkey; drfisleyen88@gmail.com; 5 Department of Paediatrics, Pediatric Neurology, Ankara University Faculty of Medicine, Ankara 06230, Turkey; patasayar@gmail.com; 6 Department of Paediatrics, Neonatology, Yildirim Beyazit University Faculty of Medicine, Ankara 06800, Turkey; dryagmur06@gmail.com (A.Y.B.); nihalelmaci@yahoo.com (N.D.)

**Keywords:** mortality, neonatal transport, TRIPS, MINT, SNAPPE-II

## Abstract

**Background:** The risk of morbidity and mortality increases in newborns requiring postpartum transport. Various scoring systems have been developed to determine mortality risk, such as the Transport Risk Index of Physiologic Stability (TRIPS) and Mortality Index for Neonatal Transportation (MINT) scores. This study aimed to evaluate the efficiency of MINT and TRIPS scores by comparing them with the Score for Neonatal Acute Physiology-Perinatal Extension (SNAPPE-II) scoring system in preterm and term infants transported within the first 24 h after birth. **Methods:** This retrospective study included neonates transported within the first 24 h of life to the NICU of Etlik Zübeyde Hanım Women’s Health Training and Research Hospital between 2016 and 2021, following ethics approval. Perinatal data, admission clinical and laboratory parameters, and TRIPS, MINT, and SNAPPE-II scores calculated within the were recorded. Mortality and short-term morbidities were analysed. Group comparisons were conducted using Mann–Whitney U and chi-square tests. Predictive performance and optimal cut-off values were determined by receiver operating characteristic curve analysis using the Youden index. *p* value <0.05 was considered significant. **Results:** A total of 137 newborns were included in the study. Seventy-two cases (52.6%) were preterm, and 65 cases (47.4%) were term newborns. The median gestational age and birthweight were 35.6 weeks and 2485 g, respectively. A total of 10 patients died. For mortality prediction, the areas under the curve for TRIPS, MINT, and SNAPPE-II were 0.919, 0.907, and 0.973, respectively (*p* < 0.001). The determined cut-off values for TRIPS, MINT, and SNAPPE-II were >19, >4, and >35, respectively. The TRIPS score showed the best accuracy for prediction of mortality in preterm infants. **Conclusions:** Our data show that MINT and TRIPS scores are efficient beyond SNAPPE-II. They demonstrated high diagnostic effectiveness in predicting mortality in preterm and term infants. The TRIPS score exhibits superior mortality prediction in preterm infants.

## 1. Introduction

Advances in neonatology have increased the chances of survival for critically ill neonates, but not every neonate may be born in the most appropriate centre. There may be a need for rapid, efficient, and safe transfer of neonates requiring tertiary and quaternary healthcare services. Although antenatal transport is the safest known transfer method, not every baby is fortunate enough to receive it. Once stabilised, the intensive care process for a neonate who has been safely transported is considered to have actually begun during the transfer [1].

Neonates requiring transport after birth are at risk for mortality and morbidity due to transport complications [2,3].

Being able to predict the risk of mortality and morbidity in patients transported to the neonatal intensive care unit facilitates patient management [1].

Various scoring systems have been developed to predict mortality and morbidity in neonatal transport. The Transport Risk Index of Physiologic Stability (TRIPS) provides guidance on the mortality of transported newborns during the first seven days [4,5].

The Mortality Index for Neonatal Transportation (MINT) scoring system consists of seven physiological parameters with a maximum score of 40. If the score is above 20, it is predicted that mortality will be high during and after transportation [6].

The SNAPPE-II score, calculated within the first 24 h after admission in newborns admitted to intensive care, is recognised worldwide for its ability to predict mortality and is one of the most widely used scoring systems in the field of neonatology globally [7].

Neonatal transportation scoring systems can assess risk before and after transportation, improve transportation efficiency, and indicate prognosis and mortality [8,9]. However, the effectiveness of neonatal transfer scores in predicting mortality risk varies [9,10,11].

This study aimed to determine the TRIPS and MINT scores of newborns transported within the first 24 h of life and to evaluate them using the SNAPPE-II score, thereby predicting short-term mortality and morbidity and determining which scoring system would be more effective in term and preterm infants.

## 2. Material and Methods

After obtaining approval from the ethics committee (22.02.2022/03), data on neonates between 0 and 24 h old transported to the Neonatal Intensive Care Unit, Etlik Zübeyde Hanım Women’s Health Training and Research Hospital by the Patient Transfer Unit of the Ministry of Health of the Republic of Turkey between 1 January 2016 and 31 December 2021 were retrospectively recorded. Gestational age, birth weight, mode of delivery, APGAR score, gender, postnatal age at the time of transfer, diagnosis, indication for transfer, clinical condition, vital signs, blood gas parameters measured within the first hour of admission to the neonatal intensive care unit, TRIPS, MINT and SNAPPE-II scores, length of stay, short-term morbidities and mortalities such as retinopathy of prematurity (ROP), necrotising enterocolitis (NEC), intraventricular haemorrhage, and sepsis were recorded.

The diagnosis of sepsis is based on general deterioration, apnoea, tachycardia, bradycardia, and an increase in acute phase reactants, or the isolation of the causative microorganism in sterile body fluids. The diagnosis of NEC was made based on abdominal distension, loss of bowel movements, abdominal tenderness, and supporting radiological findings. The diagnosis of ROP was made based on retinal vascularisation and its grading, as determined by an eye examination [12,13,14].

### 2.1. Inclusion Criteria

Patients were included in the study if they met all of the following criteria:

- Inter-hospital transport within the first 24 h after birth;
- Admission to our neonatal intensive care unit within the first 24 postnatal hours;
- Complete and accessible medical records.

### 2.2. Exclusion Criteria

Patients were excluded from the study if any of the following conditions were present:

- Missing parameters required for the calculation of TRIPS, MINT, or SNAPPE-II scores;
- Incomplete laboratory, diagnostic, or clinical examination records;
- No history of interhospital transport after birth;
- Admission to the neonatal intensive care unit later than 24 h postnatally.

Patients meeting any of these criteria were excluded from the analysis.

The patient being syndromic is not an exclusion criterion. This is because one of the questions in the MINT scoring assesses whether the patient is syndromic.

Patients born below 37 weeks of gestation are recorded as premature, while infants born at 38 weeks of gestation or above are recorded as term neonates.

Scoring was performed by the neonatologist who provided the initial intervention upon admission, within the first hour of the patient’s admission. However, as the patient’s gestational age and weight were known prior to transfer, assessments could not be performed blindly.

### 2.3. Patient Transportation System in Turkey

In our country, neonatal transports between healthcare institutions are carried out by the Ministry of Health’s land and air ambulances, which are affiliated with the 112 General Directorate operating under the Ministry of Health’s Provincial Health Directorates Emergency Health Services Department. The system used for newborn transfers is an electronic messaging/telephone call (On-Call) system (three-way). Accordingly, the physician wishing to perform a newborn transfer sends a patient transfer request form via the hospital data processing management system. The healthcare personnel/team on duty at the command centre first find a hospital, and then the 112 regional land/air ambulance carries out the transfer accompanied by a physician, depending on the healthcare personnel/team infrastructure and the patient’s clinic.

## 3. Scoring Systems

The Transport Risk Index of Physiologic Stability (TRIPS) score includes parameters such as the newborn’s body temperature, presence of respiratory distress, systolic blood pressure, and response to stimuli, and provides guidance on the mortality of transported newborns during the first seven days. The estimated mortality rate is 1% for patients with a total TRIPS score of 0–7, 3% for those with a score of 8–16, 5% for those with a score of 17–23, 15% for those with a score of 24–30, and 18% for those with a score of 31–38 [4,5].

The Neonatal Transport Mortality Index (MINT) scoring system has parameters including blood gas pH value, postnatal age, 1 min APGAR score, birth weight, PaO_2_ pressure, presence of congenital anomalies, and need for intubation. A score of 20 points or above was found to be predictive of mortality [6].

SNAPPE-II scores are evaluated based on mean arterial pressure, body temperature, PaO_2_/FiO_2_ ratio, pH value, presence of seizures, urine output, APGAR score, birth weight, and birth weight relative to gestational age [6,7]. Patients’ SNAPPE-II scores were performed within the first hour of admission to the clinic. However, the postnatal hours at the time of admission varied among patients. Therefore, for all patients the cut-off hour for SNAPPE-II was set at the first 24 h.

Patients were compared according to their scores, being term and preterm, and those discharged/transported and mortality.

## 4. Statistical Analysis

Research data were analysed using IBM SPSS Statistics 25.0 software. Normality of the data was checked with Kolmogorov–Smirnov test. Descriptive statistics were presented as median, interquartile range (IQR), number, and percentage (%). Mann–Whitney U and Chi-square tests were used for intergroup comparisons. Optimal cut-off values for TRIPS, MINT, and SNAPPE-II scores between the preterm and term groups and the discharged/exitus groups were determined using receiver operating characteristic curve analysis (ROC) based on the Youden index, which maximises the sum of sensitivity and specificity. The level of statistical significance was set at *p* < 0.05 for all analyses.

## 5. Results

A total of 137 patients were included in the study. Diagnoses of patients were prematurity (*n* = 5), perinatal asphyxia (*n* = 25), respiratory distress (*n* = 28), primary persistent pulmonary hypertension (*n* = 2), pneumothorax (*n* = 6), neonatal convulsion (*n* = 2), intrauterine growth restriction (IUGR, *n* = 2), infant of diabetic mother (*n* = 7), and Trisomy 18 (*n* = 1).

The median time of clinical admission was 5 (3–6) h. The median gestational age and birth weight were 35.6 (32–39) weeks and 2485 g, respectively. In total, 52.6% of patients were preterm, while 47.4% of them were term. One hundred and fifteen (85.2%) patients were transported from within Ankara province.

Twenty-nine patients were intubated before transport (21.2%), while 55 (%43.3) neonates needed non-invasive ventilation during transport. Twenty-six (19.1%) patients did not require oxygen during transport. Data for preterm (*n* = 65) and term (*n* = 72) newborns are shown in Table 1 and Table 2.

There were a total of 127 patients in the discharged/transported patient group (group 1), and those who died were classified as group 2.

While 124 patients were discharged, four patients were transported including two patients with cyanotic congenital heart disease (transposition of great arteries and hypoplastic left heart syndrome), one patient with perforated necrotising enterocolitis, and one patient with hypoxic-ischaemic encephalopathy.

The average day of mortality was 2.8 (1–6) days.

The median APGAR scores at 1 and 5 min were 7/8 in group 1 and 1.5/5 in group 2 (*p* = 0.001). The median body temperature was 36.5 °C in group 1 and 36 °C in group 2 (*p* = 0.013). The median hospitalisation period was 6 days in group 1 and 2 days in group 2 (*p* < 0.001). The median TRIPS score was 6 in group 1 and 41.5 in group 2 (*p* < 0.001). The median MINT score was 0 (0) in group 1 and 15.5 in group 2 (*p* < 0.001). The median SNAPPE-II score was 13 in group 1 and 75 in group 2, with a significant difference between groups (*p* < 0.001) (Table 3).

PAO_2_/FIO_2_ was significantly higher in the (*p* = 0.029). The blood gas pH level was lower in group 2 (*p* < 0.001). The days on oxygen therapy and duration of hospitalisation were significantly different between the mortality and group 1 (*p* < 0.001). Among short-term morbidities, NEC and ROP were present in one patient each in group 1 while group 2 had none. One of the five patients diagnosed with sepsis was transported with a diagnosis of perforated NEC, and one preterm infant died due to sepsis. The other three patients were discharged after recovery.

The difference between group 1 and group 2 in terms of intubation before transportation was significant (16.5% vs. 80%, *p* < 0.001). The rate of severe respiratory distress was 90% in group 2 and 9.4% in group 1 (*p* < 0.001). All patients in group 2 received MV support, while this rate was 18.1% in group 1 (*p* < 0.001) (Table 4).

**Figure 1:**
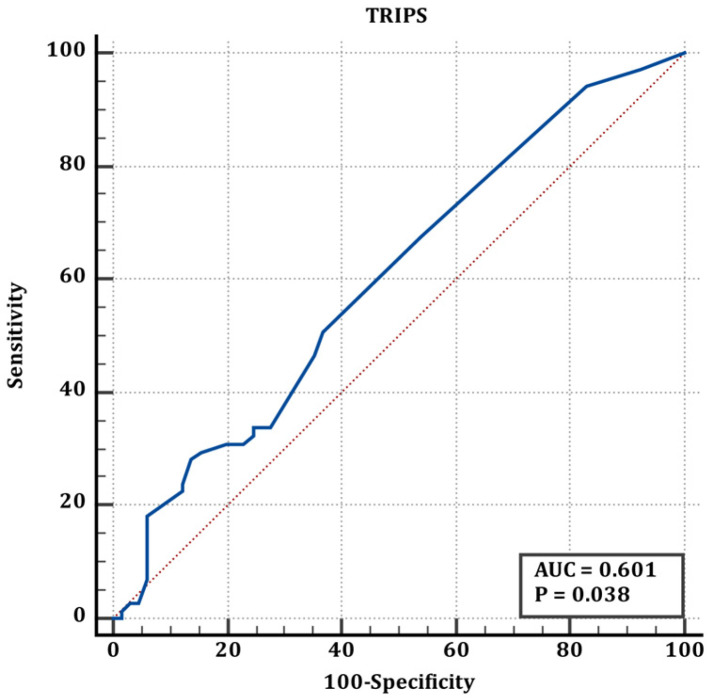
ROC curve of TRIPS score for discrimination between term and preterm neonates (corresponding to Table 5).

**Figure 2:**
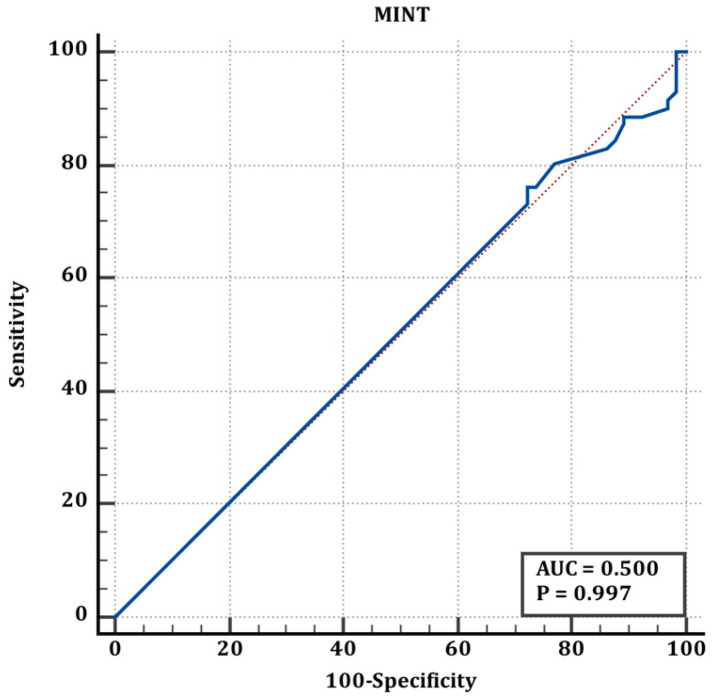
ROC curve of MINT score for discrimination between term and preterm neonates (corresponding to Table 5).

**Figure 3:**
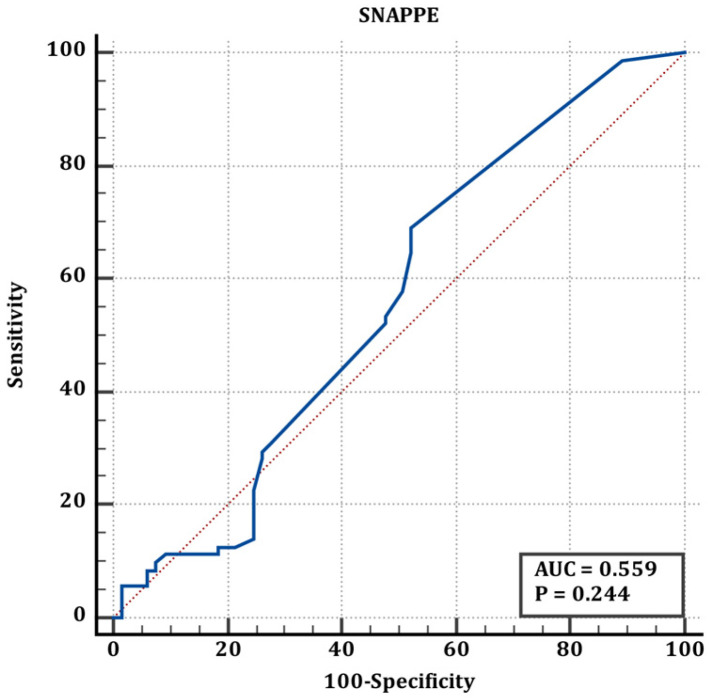
ROC curve of SNAPPE-II score for discrimination between term and preterm neonates (corresponding to Table 5).

Cut-off values of scores for preterm and term infants are shown in Table 5. According to the values shown in Table 5, the ROC curve of the TRIPS score in determining the difference between full-term and premature newborns is shown in Figure 1, the ROC curve of the MINT score in determining the difference between full-term and premature newborns is shown in Figure 2, and the ROC curve of the SNAPPE-II score in determining the difference between full-term and premature newborns is shown in Figure 3.

**Figure 4:**
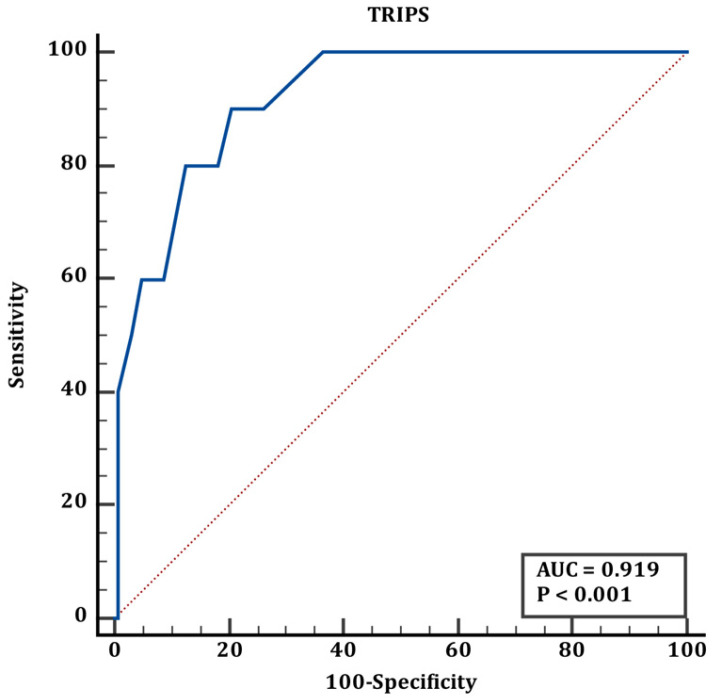
ROC curve of TRIPS score for mortality prediction in transported neonates (corresponding to Table 6).

**Figure 5:**
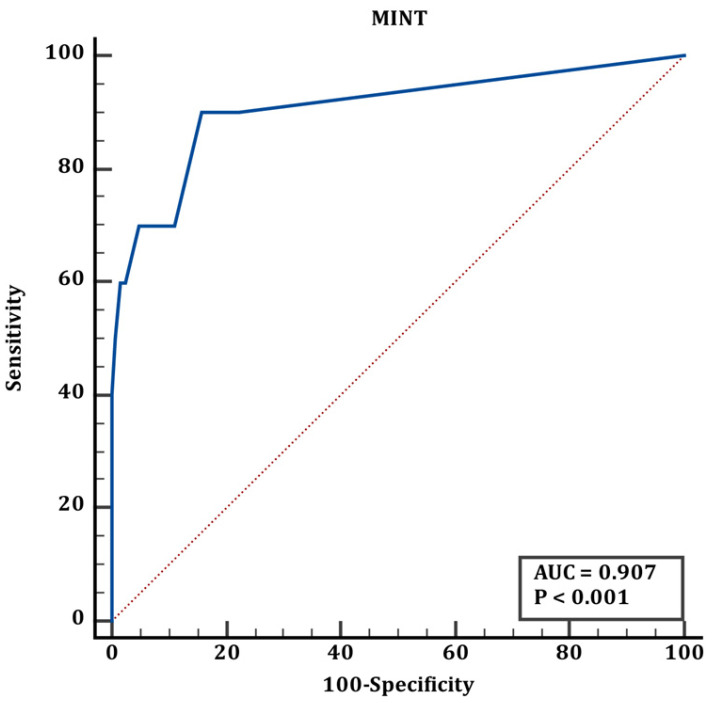
ROC curve of MINT score for mortality prediction in transported neonates (corresponding to Table 6).

**Figure 6:**
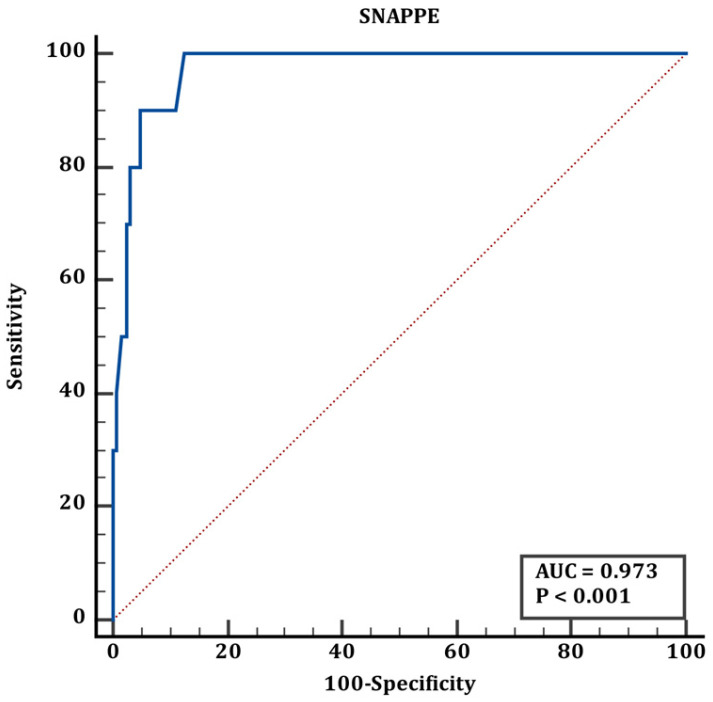
ROC curve of SNAPPE score for mortality prediction in transported neonates (corresponding to Table 6).

Cut-off values for group 1 and group 2 are shown in Table 6. According to the results shown in Table 6, the ROC curve of the TRIPS score in predicting mortality in transplanted newborns is shown in Figure 4, the ROC curve of the MINT score in predicting mortality in transplanted newborns is shown in Figure 5, and the ROC curve of the SNAPPE score in predicting mortality in transplanted newborns is shown in Figure 6.

The blue line represents the ROC curve of the scoring system, and the red line represents the reference line of no discrimination (AUC = 0.50)

## 6. Discussion

Advances in the area of neonatology over the years have increased the survival rate of critical and high-risk newborns. However, critical patient follow-up cannot be performed in every unit. Therefore, although neonates with antenatal diagnosis requiring treatment in a tertiary unit are usually transported intrauterine, postnatal transfers may sometimes be necessary due to unexpected problems. This study evaluated the mortality risk by separately assessing the transfer scoring points of patients admitted to the tertiary neonatal intensive care unit over five years. We found that the TRIPS, MINT, and SNAPPE-II scores showed positive correlation with each other and that all three provided important information in terms of mortality prediction while TRIPS was found superior in preterm infants. Years of ongoing research have demonstrated that inadequate stabilisation before transfer and inadequate care during transfer are significant risk factors for neonatal mortality and morbidity [15,16,17].

Considering that 40% of neonatal deaths occur on the first day of life and 75% within the first seven days of life, it is clear that particular attention must be paid to certain factors before and during the transport of high-risk neonates from one centre to another [17]. Neonatal transport scores can assess not only the risk of death during transport, but also the risk of death after admission to the NICU. Inter-institutional neonatal transport is the initial step in NICU treatment, and good stabilisation before transport is considered an important determinant of outcome for neonates [18]. A specialised neonatal transport system can reduce complications that may arise during transport and improve survival rates. The need to stabilise the newborn before transport and to improve neonatal care and the neonatal transport system during transport has been actively implemented in many countries, serving as a guide for clinicians [19,20].

In our country, newborn transfers are carried out in accordance with the recommendations of the “guideline on the safe transfer of newborn” prepared by the Turkish Neonatology Society [1].

Gestational age and birth weight are the most important risk factors for neonatal mortality. Although many studies evaluate transport scores separately for term and preterm infants, transport scores may vary between countries and units [21,22]. When term and preterm infants were evaluated in terms of mortality, our study showed a significant result when the threshold values for scoring systems were set at >21 for TRIPS, whereas MINT and SNAPPE-II were not statistically significant in preterm infants. TRIPS > 21 can be considered a mortality predictor for preterm infants. In a study conducted in China in 2009 by Chen et al. [23], when TREMS, MINT, and TRIPS scores were used to assess mortality rates within the first seven days in very low birth weight infants, it was found that MINT ≥ 8, TREMS ≥ 2, or TRIPS ≥ 20 could significantly increase the mortality risk of ELBW/VLBW infants within 7 days after delivery. For all infants, a TRIPS > 20 was considered an increased mortality risk, such as in our study [23].

In a recent study, Selvaraj et al. reported that the median scores for patients who died within the first seven days post-transport were 33.5, 28, 8, 3, and 7 for SNAPPE II, TRIPS, MINT, TREMS, and SNS, respectively. According to these results, the SNS score was found to have the highest sensitivity in predicting mortality, while the SNAPPE-II score had the lowest sensitivity. The negative predictive value of the TRIPS score was found to be higher than that of other scoring systems. In our study, the median TRIPS score was 41.5, and an increased risk of mortality was observed in patients with a cut-off value >19 [24].

In the study conducted by Sutcuoglu et al., the mean scores for MINT, SNAPPE II, and TREMS were reported as 6.4 ± 6.3, 8.8 ± 12, and 1.3 ± 1.1, respectively. The sensitivity of the MINT score in predicting mortality was higher than that of the SNAPPE-II and TREMS; however, the negative predictive value was highest in the MINT score. Unlike this study, our study found TRIPS to be more predictive of mortality in preterm infants [25].

Qu et al. evaluated full-term outborn patients using the Physiological Stability Transport Risk Index (TRIPS), Neonatal Transport Mortality Index (MINT), Transfer-Related Mortality Score (TREMS), and Neonatal Critical Illness Score (NCIS). They found that TRIPS and MINT scores predicted mortality within the first seven days more accurately than TREMS and NCIS (AUC 0.822 and 0.827, respectively) and found this to be statistically significant [10]. In our study, the predictive value of the TRIPS score for preterm infants was more meaningful than that of the other scores analysed.

When considering the overall mortality prediction for all cases, all three scoring systems were successful. These results indicate that all three scoring methods have high mortality prediction accuracy.

In a multicentre study conducted by Vardhelli et al., the cut-off value of ≥33 for SNAPPE-II in a scoring system evaluating 669 newborns with a gestational age of 33 weeks or more in five separate neonatal intensive care units had 94% accuracy in predicting mortality [26]. In our study, the cut-off value for the SNAPPE score was determined to be >35 with a high mortality prediction irrespective of gestational age. Another study argued that SNAPPE-II is a good predictor of mortality regardless of gestational age, but that it does not have the same effect on morbidity [27].

In a study evaluating SNAPPE-II and neonatal morbidity in very small preterm infants, the development of BPD and ROP was examined in relation to SNAPPE-II scores within the first 12 h, and it was found that a one-unit increase in SNAPPE-II increased the risk of BPD by 1.04 times. They demonstrated that elevated SNAPPE-II scores increase the risk of ROP development (cut-off for ROP is 23.5, sensitivity 80%, specificity 79%) [28]. In our study, short-term morbidities such as sepsis (*n* = 5), ROP (*n* = 3), and NEC (*n* = 1) were low in number. When evaluated together with the scores, no predictive value was determined for any of the scores.

Our data indicate that the TRIPS scoring system has high diagnostic efficacy in predicting mortality in preterm infants. However, in the subgroup analysis of term and preterm neonates, the AUC values were below 0.70, indicating weak discriminative ability. Therefore, although statistically significant in some comparisons, TRIPS should not be considered a reliable standalone mortality predictor in this subgroup. Instead, it may reflect physiological instability during transport rather than direct mortality risk. This may be because the parameters assessed in the TRIPS scoring system more clearly indicate physiological stability and more effectively evaluate respiratory distress and hypothermia, which are the most significant problems in preterm infants. When all transported patients are evaluated, both transport scoring systems are successful in predicting mortality. Neonatal transport is the initial stage of the neonatal intensive care process, and transport scoring systems provide information about the clinical condition and prognosis of the transported newborn. As the SNAPPE-II score is assessed after admission to intensive care, it may be too late to understand the severity of the condition. This is where the effectiveness and importance of transport scoring systems come to the forefront.

Our hospital is located in the Turkish capital and, as a large centre with a Level 3 neonatal intensive care unit, accepts patients from all over Turkey. In this context, it is rich in patient diversity. The evaluation of patients at different gestational ages and with different diagnoses increases the reliability of the study. Scoring patients prior to transfer is important for patient transport safety. The short- and long-term outcomes of patients transferred with correct and early stabilisation are encouraging. This study has limitations. First, due to the retrospective design, no a priori sample size calculation was performed and the sample size was determined by the number of eligible neonates within the study period. To evaluate adequacy for the primary outcome, a post hoc power analysis based on ROC discrimination against AUC = 0.50 showed power > 99% for all scoring systems. Nevertheless, the limited number of deaths may reduce the precision of estimates; therefore, results are presented with 95% confidence intervals and subgroup findings should be interpreted cautiously. The study period was chosen based on the availability and completeness of electronic medical records in the institutional database. A literature review reveals that most studies on neonatal transport are planned retrospectively. The fact that transports were carried out by experienced neonatal teams, records were kept regularly, newborns admitted to the clinic were met and scored by a neonatologist, and laboratory tests were evaluated eliminates concerns arising from the retrospective nature of the study. Therefore, the retrospective nature of the study does not confuse the scoring systems. Additionally, the lack of information about prenatal risk factors for patients is another limitation of our study.

## 7. Conclusions

All three scoring systems evaluated in this study were effective in predicting neonatal mortality risk. However, TRIPS demonstrated superior predictive performance in transported preterm neonates, suggesting its particular relevance in this high-risk population.

Given the individual variability in neonatal clinical course and outcomes, early and meticulous assessment—especially within the first 24 h of life—is critical. The appropriate application of validated neonatal transport scoring systems may improve early risk stratification and clinical decision-making.

Future efforts should focus on strengthening neonatal transport systems through standardised guidelines and context-specific scoring thresholds adapted to national geographical and sociocultural conditions. Such evidence-based and system-oriented approaches may contribute substantially to reducing mortality and morbidity among transported newborns.

## Figures and Tables

**Table 1:** The demographic characteristics of the cases.

	Premature (*n* = 65)	Term (*n* = 72)	
	Median (IQR)	Median (IQR)	*p* *
Clinic admission time	4 (3)	5 (2)	0.235
Gestational week	32 (2)	39.1 (1.2)	**<0.001**
Birth weight	1650 (395)	3460 (482.5)	**<0.001**
APGAR 1 m	7 (3)	3 (4.5)	0.115
APGAR 5 m	8 (2)	5 (3.5)	**0.003**
Systolic TA	39 (20)	53 (5)	**0.033**
Body temperature	36.0 (0.4)	36.4 (1.2)	0.540
PaO_2_/FiO_2_	1 (0.86)	1 (1.15)	0.268
Blood gases PH	7.28 (0.13)	7.3 (0.08)	0.157
IMV day	2 (2)	3 (5)	0.112
NIMV day	3 (6)	2 (3.5)	0.057
Oxygen Day	7 (8)	8 (8.5)	0.057
Hospitalisation day	25 (33)	14 (6.5)	**0.002**
TRIPS score	29 (19)	21 (11)	**0.040**
MINT score	6 (8)	6 (2.5)	0.229
SNAPPE score	30 (19)	51 (40)	0.235

*: Mann–Whitney U test. TA: arterial blood pressure, IMV: invasive mechanical ventilation, NIMV: non-invasive mechanical ventilation. Statistically significant values are shown in bold letters.

**Table 2:** The clinical characteristics of the cases.

	Premature (*n* = 65)	Term (*n* = 72)	
	*n* (%)	*n* (%)	*p* **
Birth weight			
SGA	1 (1.5)	11 (15.3)	**0.008**
AGA	62 (95.4)	58 (80.6)	
LGA	2 (3.1)	3 (4.2)	
Gender			
Male	47 (72.3)	46 (63.9)	0.360
Pre-transport intubation	14 (21.5)	15 (20.8)	0.920
Severity of respiratory distress			
Mild respiratory distress	19 (29.2)	24 (33.3)	0.204
Moderate respiratory distress	35 (53.8)	33 (45.8)	
Severe respiratory distress	7 (10.8)	14 (19.4)	
Requirement for mechanical ventilation			
Oxygen	14 (21.5)	14 (19.4)	0.478
NIMV	24 (36.9)	31 (43.1)	
IMV	14 (21.5)	19 (26.4)	
Response to painful stimulus			
None	5 (7.7)	5 (7)	0.713
Mild	11 (16.9)	16 (22.5)	
Active	49 (75.4)	50 (70.4)	
Diuresis			
<0.1 mL/h	2 (3.1)	2 (2.8)	0.070
0.1–0.9 mL/h	0 (0)	4 (5.6)	
>1 mL/h	63 (96.9)	65 (91.5)	
Evidence of sepsis	3 (4.2)	2 (3.1)	1.00
Evidence of NEC	1 (1.4)	0	1.00
Evidence of ROP	3 (4.2)	0 (0)	0.280
Exitus	6 (9.3)	4 (5.6)	0.421
Referring hospital			
Intra-province transport	56 (87.5)	59 (83.1)	0.628
Out-of-province transport	8 (12.5)	12 (16.9)	

**: Chi-Squared test. SGA: Small Gestational Age, AGA: Appropriate for Gestational Age, LGA: Large for Gestational Age, NEC: Necrotizing enterocolitis, ROP: Retinopathy of Prematurity. Statistically significant values are shown in bold letters.

**Table 3:** Characteristics of discharged and exitus cases.

	Discharged/Transported Group 1 (*n* = 127)	Exitus (*n* = 10) Group 2	
	Median (IQR)	Median (IQR)	*p* *
Clinic admission time	4 (3)	6 (6)	0.448
Gestational week	36.5 (5)	34 (7)	0.086
Birth weight	2860 (1237.5)	2395 (765)	0.105
APGAR1.m	7 (1)	1.5 (5)	**0.001**
APGAR5.m	8 (2)	5 (4)	**0.001**
Systolic TA	45 (15.75)	46.5 (10)	0.471
Temperature	36.5 (2)	36 (1.5)	**0.013**
PaO_2_/FiO_2_	50 (9.75)	56 (6)	**0.029**
Blood gases PH	7.3 (0.09)	7.005 (0.49)	**<0.001**
IMV day	1.5 (3)	2 (1)	0.932
NIMV day	2 (3)	2 (0)	0.785
Oxygen day	1 (2)	0 (1)	**0.009**
Hospitalisation day	6 (9)	2 (3)	**<0.001**
TRIPS score	6 (9)	41.5 (27)	**<0.001**
MINT score	0 (0)	15.5 (12)	**<0.001**
SNAPPE score	13 (9)	75 (23)	**<0.001**

*: Mann–Whitney U test. Statistically significant values are shown in bold letters.

**Table 4:** Characteristics of discharged/transported and exitus cases.

	Discharged/Transported Group 1 (*n* = 127)	Exitus (*n* = 10) Group 2	
	*n* (%)	*n* (%)	*p* **
Gender			
Female	40 (31.5)	4 (40)	0.839
Male	87 (68.5)	6 (60)	
Pre-transport intubation	21 (16.5)	8 (80)	**<0.001**
Severity of respiratory distress			
Mild respiratory distress	43 (33.9)	0 (0)	**<0.001**
Moderate respiratory distress	67 (52.8)	1 (10)	
Severe respiratory distress	12 (9.4)	9 (90)	
Requirement for mechanical ventilation			
Oxygen	28 (22)	0 (0)	**<0.001**
NIMV	55 (43.3)	0 (0)	
IMV	23 (18.1)	10 (100)	
Invasive MV			
None	104 (81.9)	0 (0)	**<0.001**
Yes	23 (18.1)	10 (100)	
Response to stimulus			
None	5 (4)	5 (50)	**<0.001**
Mild	23 (18.3)	4 (40)	
Active	98 (77.8)	1 (10)	
Diuresis			
<0.1 mL/h	2 (1.6)	2 (20)	**0.003**
0.1–0.9 mL/h	2 (1.6)	2 (20)	
>1 mL/h	122 (96.8)	6 (60)	
Evidence of NEC	1 (0.8)	0 (0)	1.000
Evidence of ROP	3 (2.4)	0 (0)	1.000
Referring hospital			
Intra-province transport	110 (88)	5 (50)	**0.007**
Out-of-province transport	15 (12)	5 (50)	

**: Chi-Squared test. SGA: Small Gestational Age, AGA: Appropriate for Gestational Age, LGA: Large for Gestational Age, NEC: Necrotizing enterocolitis, ROP: Retinopathy of Prematurity. Statistically significant values are shown in bold letters.

**Table 5:** Premature and term neonates’ cut-off values.

Premature & Term	AUC (95% CI)	Cut Off	Sn (95% CI)	Sp (95% CI)	*p*
TRIPS	0.60 (0.51–0.68)	>21	28.17 (18.1–40.1)	86.15 (75.3–93.5	**0.036**
MINT	0.50 (0.41–0.59)	≤12	90.14 (80.7–95.9)	3.08 (0.4–10.7)	0.996
SNAPPE	0.559 (0.47–0.64)	>5	69 (56.9–79.5)	47.69 (35.1–60.5)	0.244

Statistically significant values are shown in bold letters.

**Table 6:** Discharged/transported and death neonates cut-off values.

Discharged/Transported & Death	AUC (95% CI)	Cut Off	Sn (95% CI)	Sp (95% CI)	*p*
TRIPS		>19	90 (55.5–99.7)	79.37 (72.1–86.1)	**<0.001**
MINT	0.907 (0.85–0.95)	>4	90 (55.5–99.7)	84.13 (76.6–90)	**<0.001**
SNAPPE	0.973 (0.93–0.99)	>35	100 (69.2–100)	87.3 (80.2–92.6)	**<0.001**

Statistically significant values are shown in bold letters.

## Data Availability

The data presented in this study were obtained from the hospital’s institutional database. Due to ethical and privacy restrictions, the data are not publicly available. Anonymized data may be made available from the corresponding author upon reasonable request and with permission from the relevant institutional authorities.
